# Effect of azoxystrobin on tobacco leaf microbial composition and diversity

**DOI:** 10.3389/fpls.2022.1101039

**Published:** 2023-02-01

**Authors:** Meili Sun, Hancheng Wang, Caihua Shi, Jianjun Li, Liuti Cai, Ligang Xiang, Tingting Liu, Paul H. Goodwin, Xingjiang Chen, Ling Wang

**Affiliations:** ^1^ College of Agriculture, Yangtze University, Jingzhou, Hubei, China; ^2^ Guizhou Provincial Academician Workstation of Microbiology and Health, Guizhou Academy of Tobacco Science, Guiyang, China; ^3^ College of Tropical Crops, Hainan University, Haikou, Hainan, China; ^4^ School of Environmental Sciences, University of Guelph, Guelph, ON, Canada; ^5^ Guizhou Bijie Tobacco Company, Bijie, Guizhou, China

**Keywords:** tobacco target spot, azoxystrobin, high-throughput sequencing, microbial composition, leaf microorganisms

## Abstract

Azoxystrobin, a quinone outside inhibitor fungicide, reduced tobacco target spot caused by *Rhizoctonia solani* by 62%, but also affected the composition and diversity of other microbes on the surface and interior of treated tobacco leaves. High-throughput sequencing showed that the dominant bacteria prior to azoxystrobin treatment were *Methylobacterium* on healthy leaves and *Pseudomonas* on diseased leaves, and the dominant fungi were *Thanatephorous* (teleomorph of *Rhizoctonia*) and *Symmetrospora* on healthy leaves and *Thanatephorous* on diseased leaves. Both bacterial and fungal diversity significantly increased 1 to 18 days post treatment (dpt) with azoxystrobin for healthy and diseased leaves. For bacteria on healthy leaves, the relative abundance of *Pseudomonas*, *Sphingomonas, Unidentified-Rhizobiaceae* and *Massilia* declined, while *Methylobacterium* and *Aureimonas* increased. On diseased leaves, the relative abundance of *Sphingomonas* and *Unidentified-Rhizobiaceae* declined, while *Methylobacterium, Pseudomonas* and *Pantoea* increased. For fungi on healthy leaves, the relative abundance of *Thanatephorous* declined, while *Symmetrospora*, *Sampaiozyma*, *Plectosphaerella*, *Cladosporium* and *Cercospora* increased. On diseased leaves, the relative abundance of *Thanatephorous* declined, while *Symmetrospora*, *Sampaiozyma*, *Plectosphaerella*, *Cladosporium*, *Phoma*, *Pantospora* and *Fusarium*, increased. Compared to healthy leaves, azoxystrobin treatment of diseased leaves resulted in greater reductions in *Thanatephorous*, *Sphingomonas* and *Unidentified-Rhizobiaceae*, a greater increase in *Methylobacterium*, and similar changes in *Phoma, Fusarium*, *Plectosphaerella* and *Cladosporium*. Azoxystrobin had a semi-selective effect altering the microbial diversity of the tobacco leaf microbiome, which could be due to factors, such as differences among bacterial and fungal species in sensitivity to quinone outside inhibitors, ability to use nutrients and niches as certain microbes are affected, and metabolic responses to azoxystrobin.

## Introduction

Endophyte refers to those fungi or bacteria that live inside apparently healthy tissues of plants (Xing et al., 2015). Epiphytes refer to microorganisms living on the phylloplane (leaf surface) (Arnold et al., 2000). Endophytes live biotrophically in the apoplast of leaves (Timmer et al., 1983) playing an important role in plant growth and development and resistance to biotic and abiotic stresses (Rodriguez et al., 2009). Epiphytes live saprophytically on the leaf surface, primarily in protected microhabitats, such as near leaf hairs and veins (Andrews and Harris, 2000). The phylloplane microorganisms include many potentially beneficial, pathogenic, and antagonistic microbes affecting plant health and productivity (Lindow and Brandl, 2003).

During tobacco leaf development, many endophyte genera have been reported. The predominant fungi are *Pantospora*, *Phoma*, *Pestalotiopsis*, *Phyllachora*, *Glomerella*, *Aspergillus*, *Bipolaris*, *Trichotderma* and *Preussia* (Han, 2004; Pei, 2009; Li, 2011; Feng et al., 2019), and the predominant bacteria are *Pseudomonas* and *Paenibacillus* (Magnani et al., 2013; Paola et al., 2014). Also, during tobacco leaf development, many epiphytes have been reported. Predominant epiphytic fungi include *Alternaria*, *Boeremia*, *Phoma*, *Cercospora*, *Aspergillus*, *Rhizopus* (Liu et al., 2019; Chen et al., 2020; Xiang et al., 2020a), and predominant epiphytic bacteria include *Methylobacterium* and *Pantoea* (Liu et al., 2020; Chen et al., 2021). Among these epiphytes of tobacco, *Alternata*, *Thanatephorous*, *Boeremia*, *Phoma*, *Cercospora*, *Aspergillus*, *Rhizopus*, and *Pseudomonas* are also tobacco pathogens that could cause leaf spot diseases (Shew and Lucas, 1991).

While fungicides often have a broad specificity of activity, fungi will differ in their sensitivity to fungicides, resulting in semi-selective activity (Dzhavakhiya et al., 2012). This has been observed as non-target effects when fungicides are applied. For example, foliar application of dimetachlone reduced tobacco brown spot disease caused by *Alternaria alternata*, but the population of the phylloplane bacterium *Sphingomonas* was reduced as well (Chen et al., 2021). Another example is the treatment of potato foliage with metalaxyl-m that reduced infections by *Fusarium solani*, but also increased the populations of the endophytic bacterial species, particularly, *Pseudomonas chlororaphis* and *Pseudomonas putida* (Sturz and Peters, 2007). However, such studies have been limited.

Quinone outside inhibitor (QoI) fungicides inhibit mitochondrial respiration by blocking electron transfer at the cytochrome bc1 complex (Brandt et al., 1988). QoI fungicides show a broad inhibitory spectrum against Ascomycetes, Basidiomycetes and Oomycetes (Rideout et al., 2002). QoI fungicides can also indirectly reduce bacterial and viral diseases of plants by priming plant cellular defenses and promoting plant growth (Skandalis et al., 2016). There is evidence for differences in sensitivity of fungi to QoI fungicides. A shift towards less sensitivity to trifloxystrobin in *Cercospora sojina*, which causes frogeye leaf spot of soybean, was observed based on EC_50_ values from 2007 to 2009, likely due to greater occurrence of isolates with the alternative respiration pathway to overcome inhibition by QoI fungicides (Zhang et al., 2012). Baseline EC_50_ values of *Cercospora zeae-maydis*, which causes gray leaf spot of maize, to azoxystrobin showed a 10-fold range from 0.003 to 0.031 μg/ml (Bradley and Pedersen, 2011). *Aspergillus flavus* isolates from peanut seeds in Georgia had EC_50_ values ranging more than 100-fold from 0.12 to 297.22 μg/mL azoxystrobin, and reduced sensitivity was associated with one of two single nucleotide mutations of the *cytB* gene (Ali et al., 2021). Thus, even single mutations can change the sensitivity of QoI fungicides. However, studies on sensitivities of non-target on plants are still missing.

Tobacco (*Nicotiana tabacum* L.) is an annual, solanaceous crop grown commercially for its leaves (Guo et al., 2021). China produces nearly 40% of the total global tobacco leaves and 40% of the global tobacco consumption (Wang et al., 2016). In the last two years, tobacco target spot, a destructive foliar disease caused by *Rhizoctonia solani* Kühn (Sun et al., 2022), has occurred frequently in southwest China with losses reaching more than 50% if no disease management measures were utilized. Fungicide application is the most economical and effective method for tobacco target spot management. In the last five years, the broad-spectrum QoI fungicide azoxystrobin has been used in China for tobacco disease management. It has been shown to be effective against tobacco leaf spot diseases, such as those caused by *A. alternata* (Wang et al., 2016) and tobacco stem infections, such as those caused by *R. solani* (LaMondia, 2012), but not yet against tobacco target spot. In addition, while the effects of some fungicides on tobacco microbial communities have been previously reported (Xiang et al., 2020a; Chen et al., 2021), the effect of aQoI fungicide, such as azoxystrobin, on tobacco leaf microorganisms has not yet been described.

The goals of this study were to assess the field efficacy of azoxystrobin to tobacco target spot, and to investigate its non-target effects on total leaf fungal and bacterial composition and diversity at 0, 1, 3, 9 and 18 days’ after leaf spraying. This will provide a better understanding of the shift of epiphyte and endophyte microbial populations due to this fungicide to determine if it has differential effects on the populations, perhaps indicating differences in sensitivity to a QoI fungicide during tobacco target spot epidemic.

## Materials and methods

### *In vitro* activity of azoxystrobin against *Rhizoctonia solani*

*Rhizoctonia. solani* isolates J215, J216, and J136 with wild-type sensitivity and pathogenicity to tobacco were collected in 2020 from infected tobacco leaves in Qianxi, Guizhou, China. Stock azoxystrobin solution was prepared by dissolving technical grade azoxystrobin (93% active ingredient; Syngenta Co., Shanghai, China) in methanol and then adding to autoclaved PDA that had cooled to 50°C. The final methanol concentration in the PDA was less than 1%, which had previously been shown to not affect mycelial growth or sclerotium formation of *R. solani*. The control was the same amount of methanol added to PDA without azoxystrobin. The azoxystrobin concentrations in PDA were 6.25, 12.5, 25, 50 and 100 mg L^-1^. PDA plugs with mycelial (7-mm diameter) were transferred from the edge of an actively growing culture and placed in the center of a Petri plate (9-cm-diameter) containing PDA with or without azoxystrobin. The diameters of the colonies were measured at 4 days at 25°C in darkness, which was the time that colonies of the control reached the periphery of the plate. At 50 days at 25°C in darkness, brown sclerotia were removed and weighed. The experiment was conducted twice with three replicates each. The concentration of azoxystrobin causing 50% inhibition of mycelial growth (EC_50_) was estimated from the fitted regression line of the log-transformed percentage inhibition plotted against the log-transformed fungicide concentration (Brandt et al., 1988). Percent inhibition of sclerotium formation of *R. solani* was also calculated.

### Field efficacy of azoxystrobin

In August 2020 in Qianxi, Guizhou, China (27°03’N, 106°04’E), a 0.5 ha tobacco field (average altitude of 1250 meters) with tobacco target spot was studied, which had been continuously planted with tobacco cv. Yunyan 85 for the last 3 years. Using a completely randomized block design, tobacco plants were treated with 250 g L^-1^ azoxystrobin SC (Amistar TOP; Syngenta Co., Shanghai, China) or sterile water. The azoxystrobin SC was applied in water at 0.11 a.i. Kg ha^−1^ a in early morning according to the manufacturer’s instructions using a multi-function sprayer (model: DSF01A-20-100, Guizhou Qian Fengyuan Agricultural Technology Development Co., Guizhou, China). During the experiment, the average maximum temperature was 29°C during the day, and average minimum temperature of 20°C during the night. The average relative humidity was 71%, and there were 6 rainfall events with an average rainfall of 12.5 mm event. The soil type was laterite with a pH of 5.5 ~ 6.5.

Eight tobacco plants were randomly selected with two plants per block. At 9 days post fungicide treatment (dpt), eight tobacco target spot lesions per plant were randomly marked, and lesion size diameters were measured. From this, the inhibition of azoxystrobin on lesion expansion was calculated. The disease index was measured based on a scale (1= <1%, 5 = 11-20%, 7 = 21-40%, 9= >41% diseased leaf area) (Tan, 2009). Control efficacy of azoxystrobin against tobacco target spot was then calculated.

### Microbial composition and diversity of tobacco leaves

At 0 day (before treatment) and then 1, 3, 9 and 18 dpt, tobacco leaf tissues of diseased and healthy plants that had been treated with azoxystrobin or water were cut with sterilized scissors. Three biological repeats were conducted (**Table S1**). Due to external factors, diseased tissues were not sampled at 9 and 18 dpt. Leaf samples were placed into sterile 50 mL centrifuge tubes at 4°C, and then stored at – 80°C. Total DNA was extracted from 0.5 g samples according to the instructions of the FastDNA^®^ Spin kit (MP Biochemicals, Solon, OH, USA). DNA concentration was adjusted to 30 ng μL^-1^ (Zhang et al., 2018), and DNA purity was assessed by NanoDrop ND-2000 (Thermo Fisher Scientific, Waltham MA, USA).

The bacterial hypervariable V4 region was amplified using the primers 338F (5’-ACTCCTACGGGAGGCAGCAG-3’) and 806R (5’-GGACTACHVGGGTWTCTAAT-3’) (Xiang et al., 2020b). The fungal ITS1-5F region was amplified using the primers ITS1F (5’-CTTGGTCATTTAGAGGAAGTAA-3’) and ITS2R (5’-CCTGCGTTCTTCATCGATGC-3’) (Xiang et al., 2020c). The PCR reaction (4 μL 5 × FastPfu Buffer, 2 μL 2.5 mM dNTPs, 0.8 μL each primer, 0.4 μL FastPfu Polymerase, 1 μL DNA and 11.8 μL water) was performed on a VeritiPro™ thermal cycler (Thermo Fisher Scientific). The 16S rRNA conditions were: 94 °C for 3 min, followed by 30 cycles of 94°C for 45 s, 55°C for 45 s and 72°C for 90 s, finally 7 min at 72°C. The ITS rDNA conditions were: 94°C for 5 min, followed by 35 cycles of 94°C for 1 min, 57°C for 1 min and 72°C for 1 min, finally 5 min at 72°C. PCR products were detected by 2% agarose gel eletrophoresis and purified with Gene JET (Thermo Fisher Scientific). High-throughput sequencing was performed on the Lon S5 XL platform at Novogene Bioinformatics Technology Co, Beijing, China. The corresponding length of paired-end sequencing was 250 bp according to the standard protocol.

Sequencing data was filtered and optimized by FLASH and Trimomatic software, and the paired sequences were connected. High-quality sequences were clustered into OTUs (operational taxonomic units) by UPARSE software (version 7.1) with a 97% similarity, and the singletons and chimeras were removed in the clustering process. The OTUs were annotated with SILVA 132 for the bacterial 16S rRNA and UNITE version 7.2 for the fungal ITS (Edgar and Robert, 2013; Urmas et al., 2013). The Alpha-diversity index was calculated by Qiime software (version 1.9.1), and the diversity of tobacco leaf microbial community was analyzed by Shannon index. The richness index was analyzed by the Chao 1 index and ACE index, and the Goods Coverage index was used to analyze the coverage of tobacco leaf microbial community (Xie et al., 2021). The Beta-diversity was calculated by Qiime software (version 1.9.1). IBM SPSS Statistics 23 (IBM Corp., New York, NY, USA) was used to compare the differences of Alpha-diversity indexes of fungal and bacterial communities. Fungal nutritional modes were analyzed with FUNGuild database (Nguyen et al., 2016) and bacterial metabolic functions were analyzed with bioinformatics software package PICRUSt (Langille et al., 2013). Software of Cytoscape 3.9.1 (https://cytoscape.org/) was used to evaluate the interactions among microbial community rate (Williams et al., 2014; Meng et al., 2017). The R statistics package (R Core Team, 2017) was used for Principal Co-ordinate Analysis (PCoA) (Version 1.9.1) and to create the rarefaction curves (Version 2.15.3) and Venn diagrams (Version 3.0.3).

## Results

### *In vitro* activity of azoxystrobin against mycelial growth and sclerotial formation of *Rhizoctonia solani*

The EC_50_ value of azoxystrobin against mycelial growth of three isolates *R. solani in vitro* ranged from 14.13 to 16.68 mg/L with an average of 15.71 mg/L (**Table 1**). The percent inhibition of azoxystrobin to sclerotium formation of three isolates *R. solani* was ranged from 47.09% at 6.25 mg/L to 100% at 12.5, 50 and 100 mg/L (**Table 2**).

**Table 1:** *In vitro* activity of azoxystrobin against mycelial growth of *Rhizoctonia solani* isolates J215, J216 and J36 from tobacco.

Fungicide	Isolate	Regression equation	*r*	EC_50_ (mg/L)	Average value of EC_50_(mg/L)
	J215	*y* = 1.89*x* + 2.69	0.94	16.68	15.71 ± 1.38
**azoxystrobin**	J216	*y* = 1.99*x* + 2.59	0.98	16.31	
	J136	*y* = 2.15*x* + 2.53	0.97	14.13	

**Table 2:** *In vitro* inhibition of azoxystrobin against sclerotial formation by *Rhizoctonia solani* isolates J215, J216 and J36 from tobacco.

Fungicide	Conc. / (mg/L)	Inhibition rate / %			
		J215	J216	J136	Average
**azoxystrobin**	6.25	-40.00	100.00	81.28	47.09 ± 76.00 ab
	12.5	100.00	100.00	100.00	100.00 ± 0.00 a
	25	40.00	100.00	36.36	58.79 ± 35.74 ab
	50	100.00	100.00	100.00	100.00 ± 0.00 a
	100	100.00	100.00	100.00	100.00 ± 0.00 a

Data in column followed by the same letters were not significantly different at P<0.05 level according to Duncan’s new multiple range test.

### Field efficacy of azoxystrobin against tobacco target spot

Nine days after application of azoxystrobin, the tobacco target spot lesion diameter was significantly less than that of the control (**Table 3**). The reduction in lesion diameter was 75.10%. Although the control efficacy was comparable at 61.67%, there was no significant difference in the disease index between azoxystrobin treatment and the control (**Table 3**).

**Table 3:** Field control of tobacco target spot by azoxystrobin.

Treatment	Lesion Diameter (mm)	Inhibition (%)	Disease Index	Control Efficacy (%)
**control**	4.65 ± 0.34 a	——	9.75 ± 7.69 a	——
**azoxystrobin**	1.16 ± 0.04 b	75.10	4.33 ± 0.00 a	61.76

Means followed by different letters indicate a significant difference using the Duncan’s new multiple range test (P < 0.05).

### Quality of bacterial and fungal sequence data

**Figure 1:**
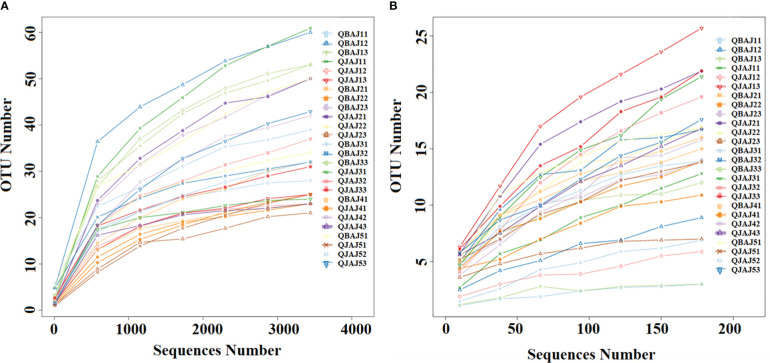
Rarefaction curves of bacterial **(A)** and fungal **(B)** OTUs across different tobacco leaf samples.

For 11 diseased samples and 15 healthy samples, a total of 706,120 and 979,573 bacterial sequences were obtained that were classified into 304 operational taxonomic units (OTUs) at a 97% similarity, and a total of 847,123 and 81,187,375 fungal sequences were obtained that were classified into 114 OTUs at 97% similarity. When the number of bacterial and fungal sequences reached 3,000 and 150, respectively, the rarefaction curves reached the plateau stage (**Figure 1**), suggesting that the sequencing depth was sufficient to reflect the structure and diversity of the communities.

### Bacterial and fungal OTU distribution and diversity

Among the bacterial OTUs, the only significant Shannon diversity index between healthy and diseased leaves was on day 3 at 0.98 and 2.43, respectively (**Table 4**). However, there was no significant difference in the Chao1 richness index and ACE richness index between healthy and diseased leaves. The coverage index was significantly difference in between healthy and diseased leaves on day 1 at 0.997 and 0.995, respectively.

**Table 4:** Alpha-diversity and coverage indices of bacterial and fungal communities before and after application of azoxystrobin based on high-throughput sequencing.

	Application time	Sample Group	Diversity index	Richness index		Coverage index
			Shannon index	Chao1 index	ACE index	Coverage
**Bacteria**	0 d	QBAJ1	1.67 ± 0.25 bc	28.95 ± 3.95 b	32.48 ± 5.13 b	0.998 ± 0.001 ab
		QJAJ1	1.79 ± 0.20 ab	40.17 ± 12.79 ab	39.60 ± 15.60 ab	0.997 ± 0.001 abc
	1 d	QBAJ2	1.03 ± 0.61 cd	52.06 ± 14.27 a	59.59 ± 12.42 a	0.995 ± 0.001 c
		QJAJ2	0.76 ± 0.42 d	37.04 ± 17.94 ab	39.50 ± 20.48 ab	0.997 ± 0.001 ab
	3 d	QBAJ3	2.43 ± 0.68 a	39.64 ± 14.90 ab	42.31 ± 13.68 ab	0.998 ± 0.001 a
		QJAJ3	0.98 ± 0.17 d	29.34 ± 8.10 b	32.02 ± 10.20 b	0.998 ± 0.001 a
	9 d	QJAJ4	0.55 ± 0.09 d	34.21 ± 9.75 ab	36.04 ± 11.48 b	0.997 ± 0.0006 ab
	18 d	QJAJ5	0.59 ± 0.32 d	44.63 ± 5.69 ab	47.18 ± 7.54 ab	0.996 ± 0.0006 bc
**Fungi**	0 d	QBAJ1	0.54 ± 0.60 c	6.67 ± 3.67 c	8.52 ± 8.61 c	0.99 ± 0.01 a
		QJAJ1	1.86 ± 1.45 a	24.75 ± 16.24 bc	27.47 ± 14.96 bc	0.96 ± 0.02 b
	1 d	QBAJ2	2.55 ± 0.33 ab	19.57 ± 3.86 bc	21.38 ± 3.47 bc	0.97 ± 0.01 ab
		QJAJ2	2.65 ± 0.60 ab	18.25 ± 9.88 bc	20.37 ± 11.45bc	0.97 ± 0.02 ab
	3 d	QBAJ3	1.87 ± 1.17 b	14.44 ± 5.32 bc	17.89 ± 3.73 bc	0.98 ± 0.01 ab
		QJAJ3	2.69 ± 0.21 ab	48.61 ± 22.90 a	69.91 ± 33.17 a	0.93 ± 0.01 c
	9 d	QJAJ4	2.48 ± 0.14 ab	23.68 ± 8.02 bc	31.12 ± 14.19 bc	0.96 ± 0.01 b
	18 d	QJAJ5	2.63 ± 0.20 ab	25 ± 13.54 bc	30.28 ± 13.96 bc	0.96 ± 0.02 b

Data in column followed by the same letters were not significantly different at P<0.05 level according to Duncan’s new multiple range test.

Analysis was not performed for results of diseased samples at 9 and 18 dpt as there was only one replication.

Among the fungal OTUs, the only significant Shannon diversity index between healthy and diseased leaves was on day 0 at 1.86 and 0.54, respectively (**Table 4**). Similarly, the only significant difference in the Chao 1 richness index between healthy and diseased leaves was on day 3 at 48.61 and 14.44, respectively. A significant difference in the ACE richness index between healthy and diseased leaves was observed on day 3 at 69.91 and 17.89, respectively. There were significant differences in the coverage index between healthy and diseased leaves at both day 0 at 0.96 and 0.99, respectively, and day 3 at 0.93 and 0.98, respectively.

### Bacterial and fungal community composition

**Figure 2:**
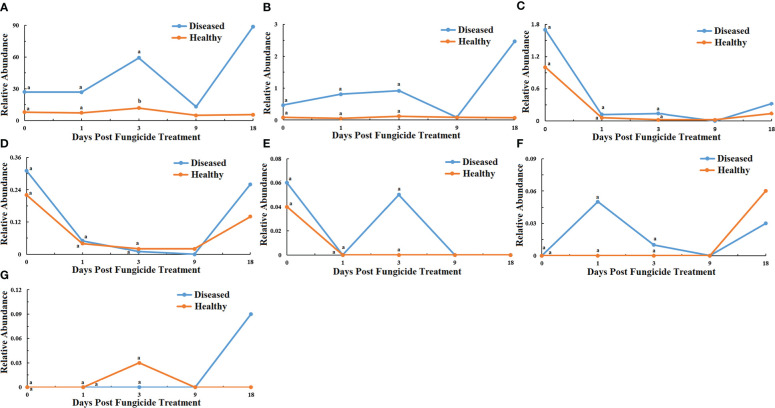
Changes in bacterial community compositions at the phylum level for diseased and healthy leaves over time after azooxystrobin treatment. **(A)** Proteobacteria, **(B)** Actinobacteria, **(C)** Firmicutes, **(D)** Bacteroidetes, **(E)** Deinococcus-Thermus, **(F)** Synergites, **(G)** Acidobacteria. Statistical analysis was not performed for results of diseased samples at 9 and 18 dpt as there was only one replication.

The 16S rRNA dataset showed that all the bacterial OTUs could be classified into seven phyla (**Figure 2**). The relative abundance of Proteobacteria was the highest among the phyla (**Figure 2A**). For healthy leaves, it was relatively consistent over time, whereas it showed peaks in abundance at 3 and 18 dpt for diseased leaves, with significantly higher abundance at 3 dpt. The next most abundant phyla were the Actinobacteria and Firmicutes but typically 10-fold less than the Proteobacteria (**Figures 2B, C**). Once again, the relative abundance of Actinobacteria on healthy leaves showed little change over time, but there was a peak at 18 dpt for diseased leaves (**Figure 2B**). Firmicutes showed a similar pattern in healthy and diseased leaves with a decline in abundance at 1 dpt (**Figure 2C**). Bacteroidetes was generally less abundant, also with a similar pattern in healthy and diseased leaves showing a decline in abundance at 1 dpt and an increase at 18 dpt (**Figure 2D**). The least abundant were the Deinococcus-Thermus, Synergistetes and Acidobacteria (**Figures 2E–G**). The Deinococcus-Thermus only were detected in healthy leaves at 0 dpt and only detected at 0 and 3 dpt in diseased leaves (**Figure 2E**). Synergistetes were only detected in healthy leaves at 18 dpt, whereas they were detected in diseased leaves at 1, 3 and 18 dpt (**Figure 2F**). The Acidobacteria were mostly undetectable, except for healthy leaves at 3 dpt and diseased leaves at 18 dpt (**Figure 2G**).

**Figure 3:**
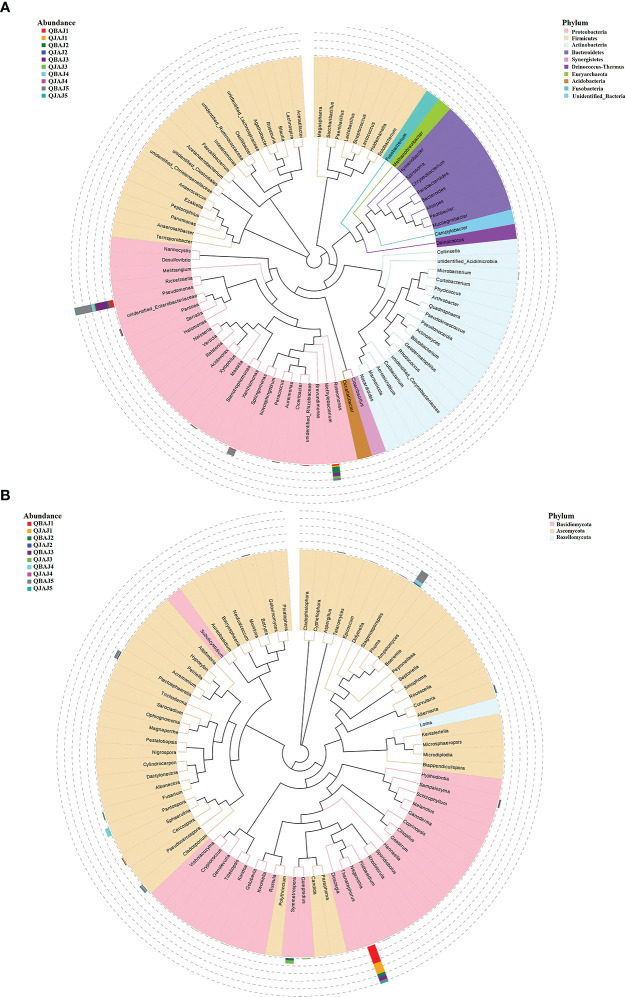
Maximum likelihood tree of the 100 most abundant bacterial **(A)** and fungal **(B)** genera in the ten group samples from tobacco leaves infected with target spot, obtained by analysis of 16S rRNA and ITS rDNA pyrosequencing data. A color-coded bar plot shows the distribution of each fungal and bacterial genus in different groups.

**Figure 4:**
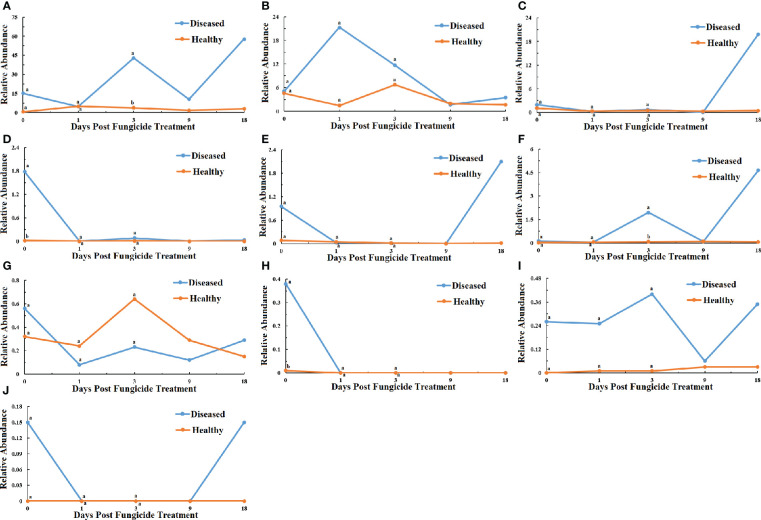
Changes in bacterial community compositions at the genus level for diseased and healthy leaves over time after azooxystrobin treatment. **(A)** *Pseudomonas*, **(B)** *Methylobacterium*, **(C)** *Sphingomonas*, **(D)** *unidentified_Rhizobiaceae*, **(E)** *Massilia*, **(F)** *Pantoea*, **(G)** *Aureimonas*, **(H)** *unidentified_Christensenellaceae*, **(I)** *Microbacterium*, **(J)** *Paenibacillus*. Statistical analysis was not performed for results of diseased samples at 9 and 18 dpt as there was only one replication.

At the genus level for bacteria, a maximum-likelihood tree of the 100 most abundant genera showed that they belonged to ten phyla with the most genera in the Proteobacteria, followed by Actinobacteria, Firmicutes, Bacteroidetes, Deinococcus-Thermus, Synergites, Acidobacteria and the fewest genera in the Euryarchacota, Fusobacteria and Unidentified-Bacteria. (**Figure 3A**). The ten most abundant genera were *Pseudomonas*, *Methylobacterium*, *Sphingomonas*, *unidentified_Rhizobiaceae*, *Massilia*, *Pantoea* and *Aureimonas* in the Proteobacteria, *Microbacterium* in the Actinobacteria, and *unidentified_Christensenellaceae* and *Paenibacillus* in the Firmicutes (**Figure 4**). The relative abundance of *Pseudomonas* for healthy leaves was relatively consistent over time, whereas it showed peaks in abundance at 3 and 18 dpt for diseased leaves, with significantly higher abundance at 3 dpt (**Figure 4A**). Similarly, the relative abundance of *Methylobacterium* on healthy leaves showed little change over time, but there was a peak at 1 dpt for diseased leaves (**Figure 4B**). *Sphingomonas* abundance showed a similar pattern in healthy and diseased leaves with low levels, except for a peak at 18 dpt for diseased leaves (**Figure 4C**). The *unidentified_Rhizobiaceae* were not detectable in healthy leaves, but were detectable in diseased leaves at 1 dpt and to a much lesser extent at 3 dpt (**Figure 4D**). *Massilia* were detectable in healthy leaves only at 0 dpt, but were detectable in diseased leaves at 0 and 18 dpt (**Figure 4E**). *Pantoea* were not detected in healthy leaves but were detected in diseased leaves at 3 and 18 dpt with a significant difference at 3 dpt between healthy and diseased leaves (**Figure 4F**). *Aureimonas* abundance fluctuated over time in both healthy and diseased leaves (**Figure 4G**). The *unidentified_Christensenellaceae* was only detectable in healthy and diseased leaves at 0 dpt (**Figure 4H**). *Microbacterium* was detectable at low levels in healthy leaves, except at 0 dpt, whereas it was detectable at all time points in diseased leaves at higher abundances (**Figure 4I**). *Paenibacillus* were not detectable in healthy leaves, but were detectable in diseased leaves at 0 and 18 dpt (**Figure 4J**).

**Figure 5:**
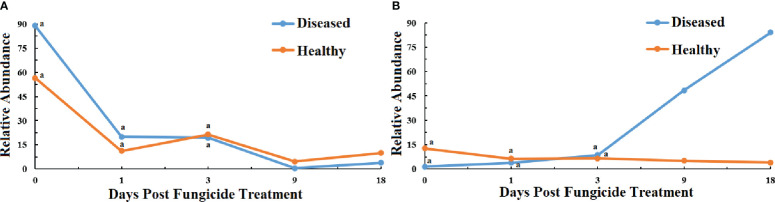
Changes in fungal community compositions at the phylum level for diseased and healthy leaves over time after azooxystrobin treatment. **(A)** Basidiomycota, **(B)** Ascomycota. Statistical analysis was not performed for results of diseased samples at 9 and 18 dpt as there was only one replication.

The ITS rRNA dataset showed that all the fungal OTUs could be classified in two phyla (**Figure 5**). The relative abundance of Basidiomycota showed a similar pattern in healthy and diseased leaves with a decline in abundance at 1 dpt (**Figure 5A**). The abundance of Ascomycota for healthy leaves showed little change over time, but there was a peak at 18 dpt for diseased leaves (**Figure 5B**).

**Figure 6:**
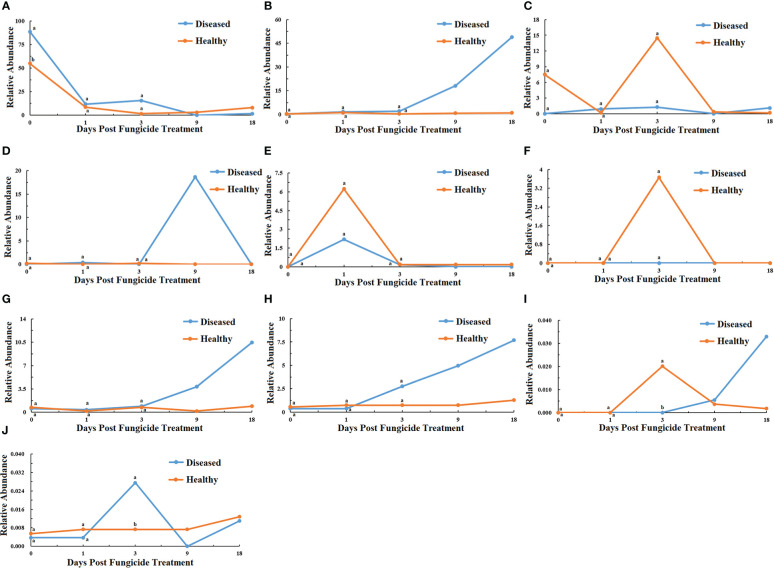
Changes in fungal community compositions at the genus level for diseased and healthy leaves over time after azoxystrobin treatment. **(A)** Thanatephorus, **(B)** Phoma, **(C)** Symmetrospora, **(D)** Pantospora, **(E)** Cercospora, **(F)** Gomphidius, **(G)** Plectosphaerella, **(H)** Cladosporium **(I)** Fusarium, **(J)** Sampaiozyma. Statistical analysis was not performed for results of diseased samples at 9 and 18 dpt as there was only one replication.

At the genus level for fungi, a maximum-likelihood tree of the 100 most abundant genera showed that the Basidiomycota being the most common, followed by Ascomycota with only one genus in the Rozellomycota (**Figure 3B**). All the ten most abundant genera, *Thanatephorus*, *Sampaiozyma*, *Symmetrospora*, *Gomphidius* in the Basidiomycota, and *Phoma*, *Pantospora*, *Cercospora*, *Plectosphaerella*, *Cladosporium* and *Fusarium*, were in the Ascomycota (**Figure 6**). The relative abundance of *Thanatephorus* for healthy leaves and diseased leaves showed a similar pattern with a decline in abundance at 1 dpt (**Figure 6A**). *Phoma* were not detectable in healthy leaves, but were detected in diseased leaves with a peak abundance at 18 dpt (**Figure 6B**). The relative abundance of *Symmetrospora* on healthy leaves showed a peak at 3 dpt, but little change over time on diseased leaves (**Figure 6C**). *Pantospora* was not detectable in healthy leaves but was detectable in diseased leaves at 1 and 9 dpt with a peak at 9 dpt (**Figure 6D**). *Cercospora* showed a similar pattern in healthy and diseased leaves with a peak at 1 dpt (**Figure 6E**). *Gomphidius* was detectable in healthy leaves at 3 dpt but not detectable in diseased leaves (**Figure 6F**). Both *Plectosphaerella* and *Cladosporium* abundance showed a similar pattern with little change over time in healthy leaves but increasing over time in diseased leaves (**Figures 6G, H**). *Fusarium* for healthy leaves and showed a peak in abundance at 3 dpt, which was significantly greater than for diseased leaves, and a peak in abundance at 18 dpt for diseased leaves (**Figure 6I**). *Sampaiozyma* abundance in healthy leaves was relatively constant over time whereas diseased leaves showed a peak at 3 dpt, which was significantly higher than that of healthy leaves (**Figure 6J**).

### Distribution of bacterial and fungal and communities between leaves

**Figure 7:**
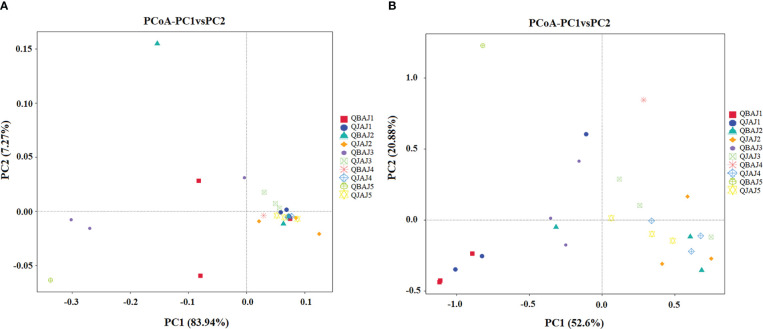
Principal Co-ordinate Analysis (PCoA) analysis of the bacterial **(A)** and fungal **(B)** communities in the different group samples.

Principal Co-ordinate Analysis (PCoA) was used to examine whether the distribution of the bacterial and fungal communities differed between healthy or diseased leaves between samples. For bacteria, samples from diseased leaves at 0, 1 and 3 dpt were slightly separated from the other samples (**Figure 7A**). For fungi, samples from healthy leaves at 0 dpt and diseased leaves at 1 dpt were slightly separated from the others (**Figure 7B**).

### Bacterial and fungal functional characteristics

**Figure 8:**
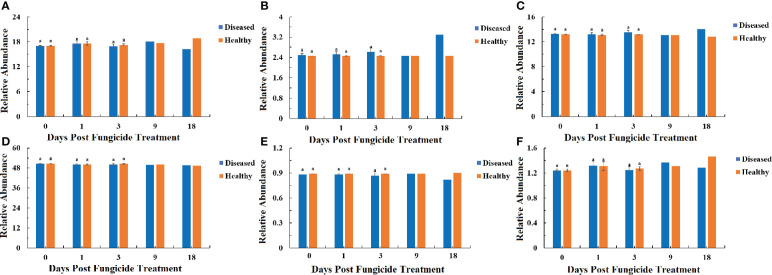
PICRUSt analyses of the changes in the KEGG level 1 functional categories of bacteria in tobacco leaf samples. **(A)** Genetic Information Processing, **(B)** Cellular Processes, **(C)** Environmental Information Processing, **(D)** Metabolism, **(E)** Organismal Systems, **(F)** Human Diseases. Statistical analysis was not performed for results of diseased samples at 9 and 18 dpt as there was only one replication.

**Figure 9:**
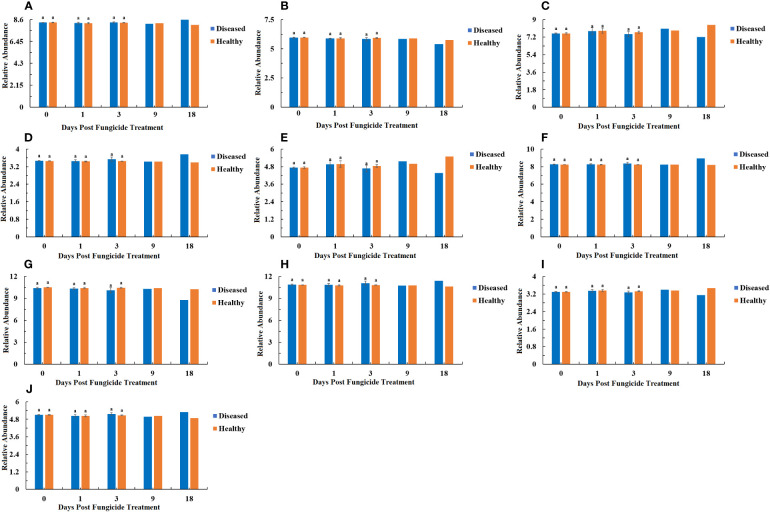
PICRUSt analyses of the changes in the KEGG level 2 functional categories of bacteria in tobacco leaf samples. **(A)** Carbohydrate Metabolism, **(B)** Metabolism of Cofactors and Vitamins, **(C)** Replication and Repair, **(D)** Cellular Processes and Signaling, **(E)** Translation, **(F)** Amino Acid Metabolism, **(G)** Energy Metabolism, **(H)** Membrane Transport, **(I)** Nucleotide Metabolism, **(J)** Poorly Characterized. Statistical analysis was not performed for results of diseased samples at 9 and 18 dpt as there was only one replication.

**Figure 10:**
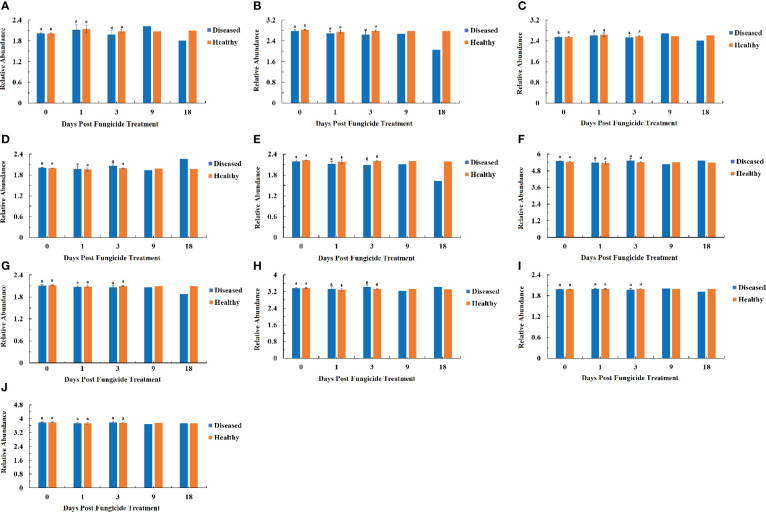
PICRUSt analyses of the changes in the KEGG level 3 functional categories of bacteria in tobacco leaf samples. **(A)** Ribosome, **(B)** Photosynthesis proteins, **(C)** DNA repair and recombination proteins, **(D)** Two component system, **(E)** Photosynthesis, **(F)** Peptidases, **(G)** Transporters, **(H)** ABC transporters, **(I)** Purine metabolism, **(J)** General function prediction only. Statistical analysis was not performed for results of diseased samples at 9 and 18 dpt as there was only one replication.

The bacterial community functions differed between samples based on level 1, 2 and 3 categories in the Kyoto Encyclopedia of Genes and Genomes (KEGG) pathways (**Figures 8**–**10**). The relative abundance of the level 1categories of the KEGG pathways for diseased and healthy leaves fluctuated over time, but with no clear patterns, except for an increase in cellular processes in diseased leaves at 18 dpt (**Figure 8**). This was similar for level 2 categories, although there was a slight decline in diseased leaves at 18 dpt for metabolism of cofactors and vitamins, replication and repair, translation, energy metabolism, and nucleotide metabolism (**Figure 9**). Similar results were observed for level 3 categories with a slight decline in diseased leaves at 18 dpt for ribosome, photosynthesis proteins, DNA repair and recombination proteins, photosynthesis, and transporters (**Figure 10**). However, there were no significant differences between healthy and diseased leaves at any time point for any category.

**Figure 11:**
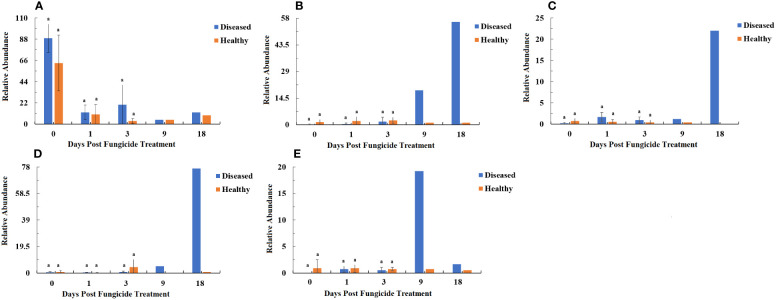
Relative abundance of fungal functional groups (modes) based on OTU annotation table with disturbance frequency level. **(A)** Pathotroph, **(B)** Pathotroph-Saprotroph, **(C)** Pathotroph-Saprotroph-Symbiotroph, **(D)** Pathotroph-Symbiotroph, **(E)** Saprotroph. Statistical analysis was not performed for results of diseased samples at 9 and 18 dpt as there was only one replication.

The fungal community trophic modes were analyzed with the FUNGuild database (**Figure 11**). The relative abundance of pathotroph mode in healthy and diseased leaves showed a major drop after 0 dpt (**Figure 11A**). The relative abundance of pathotroph-saprotroph mode in healthy leaves was relatively constant over time, but in diseased leaves it showed increases at 9 and 18 dpt (**Figure 11B**). The relative abundance of pathotroph-saprotroph-symbiotroph mode also showed little change over time in healthy leaves, but an increase at 18 dpt in diseased leaves it fluctuated over time but with no clear pattern and had a peak at 18 dpt (**Figure 11C**). The pattern of the relative abundance of pathotroph-symbiotroph mode over time in healthy and diseased leaves was similar to that of the pathotroph-saprotroph-symbiotroph mode (**Figure 11D**). The relative abundance of saprotroph mode in healthy leaves was relatively consistent over time, but in diseased leaves, there was a large increase at 9 dpt (**Figure 11E**). There were no significant differences between healthy and diseased leaves at any time point for any mode.

### Bacterial and fungal co-occurrence

**Figure 12:**
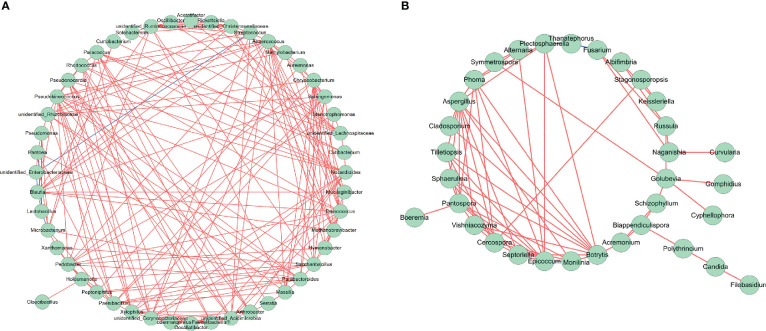
Network analysis of bacterial **(A)** and fungal **(B)** taxa showing significant positive or negative co-occurrences based on Spearman’s rank correlations. Red lines represent positive correlations and blue lines represent negative correlations.

Network analysis of the co-occurrence of the most abundant 50 bacterial species revealed that *Pseudomonas*, *Pantoea* and *Microbacterium* showed a significant negative co-occurrence with *Lactobacillus* (P < 0.05, r < -0.4), and *unidentified-Enterobacteriaceae* showed a significant negative co-occurrence with *Blautia* and *Streptococcus* (P < 0.05, r < -0.4). All 50 bacterial species showed positive co-occurrence with at least one other bacterial species (P < 0.05. r > 0.4) (**Figure 12A**). Network analysis of the co-occurrence of the most abundant 50 fungal species revealed that *Thanatephorous* showed a highly significant negative co-occurrence with *Plectosphaerella* and *Fusarium* (P < 0.05, r < -0.4). There were 49 fungal species showing positive co-occurrence with each other (P < 0.05. r > 0.4) (**Figure 12B**). The only fungal species not to show a positive co-occurrence with at least one other fungal species was *Thanatephorous*.

## Discussion

### Control of target spot by azoxystrobin

Azoxystrobin has been shown to control *R. solani* infections in a number of crops. In sugar beets, there was a 83.3% reduced leaf lesion area caused by *R. solani* with seed applied 22.9% azoxystrobin at 0.17 kg a. i ha^-1^ (Khan et al., 2017). For rice, foliarly applied 25% azoxystrobin at 0.19 kg a. i ha^-1^ reduced *R. solani* leaf lesion areas by 77% (Chen et al., 2015). Leaf lesion area of peanuts caused by *R. solani* was reduced 58.9% by foliarly applied 25% azoxystrobin at 0.09 kg a. i ha^-1^ (Paredes et al., 2021). For tobacco, foliarly applied 22.9% azoxystrobin at 0.19 kg a. i ha^-1^ resulted in an 86.77% reduction in stem lesion area due to *R. solani* (Wang et al., 2016). In this study, tobacco leaf lesion size caused by *R. solani* was reduced by 75.10% after application of 25% azoxystrobin at 0.11 a.i. Kg ha^-1^. Thus, the efficacy of azoxystrobin was comparable to that of many other studies but slightly lower than that in some studies. In addition, there was also no evidence for phytotoxicity by azoxystrobin in this study. No phytotoxicity has also been reported in studies on other crops where azoxystrobin was applied as a foliar spray (Chen et al., 2015; Wang et al., 2016; Khan et al., 2017).

### Leaf endophytes versus epiphytes

The samples collected in this study came from entire leaves, and thus would contain both endophytes and epiphytes. A literature search of the ten most abundant bacteria in this study showed that five were previously described as endophytes and five as epiphytes (**Table S2**). For the ten most abundant fungi in this study, eight have been described as endophytes and two as epiphytes (**Table S2**). However, some of those organisms would be found both in the phyloplane and endosphere as many leaf endophytes first colonize the phylloplane and then enter the leaves through wounds and natural openings (Praca et al., 2012). The diversity of endophytes is lower than that of epiphytes as not all epiphytes are adapted to grow as endophytes (Bulgarelli et al., 2013). In healthy azoxystrobin-treated leaves, changes over time of the ten most abundant bacteria showed that the populations of the five bacteria described as endophytes (**Table S2**) generally remained unchanged, except for *Pseudomonas* and *Microbacterium*, which showed peaks at 3 and 18 dpt, and the five bacteria described as epiphytes (**Table S2**) generally remained unchanged, except for *Methylobacterium* and *Aureimonas*, both of which showed a peak at 3 dpt. Changes in abundance over time of the ten most abundant fungi showed that the two fungi described as endophytes (**Table S2**) either remained unchanged or had a peak at 3 dpi, whereas the eight fungi described as epiphytes (**Table S2**) were either unchanged over time or showed peaks at 0 or 3 dpt. Based on this limited sample, one can conclude that the abundances of endophytes or epiphytes were not notably different in response to azoxystrobin in healthy leaves.

### Tobacco leaf microbiome prior to azoxystrobin application

The most dominant genera of bacteria in healthy tobacco leaves prior to azoxystrobin treatment was *Methylobacterium*, whereas it was *Pseudomonas* in diseased leaves. Identification of the bacterial species from the tobacco phyllosphere based on 16S rRNA sequences of DGGE bands showed that four of the nine bacteria were *Pseudomonas* (Lv et al., 2012). Other studies of the tobacco leaf microbiome showed that the most abundant bacteria were *Pseudomonas* and *Pantoea* for healthy and brown spot infected tobacco leaves (Liu et al., 2020), and *Pseudomonas* and *Kosakonia* for healthy and brown spot infected tobacco leaves prior to application of Bordeaux mixture (Liu et al., 2022). *Pantoea* was among the ten most dominant genera of bacteria in tobacco leaves in this study, but only after azoxystrobin treatment in diseased leaves. *Kosakonia* was among the 50 most abundant bacteria in this study, but not among the top ten. The diversity of bacteria for healthy leaves prior to azoxystrobin treatment was not significantly higher than that of diseased leaves. In a study comparing healthy and *Boeremia* infected tobacco leaves, the diversity of bacteria in healthy leaves was higher than that of in diseased leaves (Xiang et al., 2021). However, in a study comparing healthy and bacterial shank infected tobacco stems, the diversity of bacteria in healthy stems was lower than that of diseased stems (Xiang et al., 2020b). Further work is needed to conclude whether pathogen infections more often reduce or increase bacterial diversity in tobacco.

The most dominant genera of fungi with healthy leaves prior to azoxystrobin treatment were *Thanatephorous* (teleomorph of *R. solani*) and *Symmetrospora*, whereas in diseased leaves it was *Thanatephorous.* This was not surprising for diseased leaves as *R. solani* was the pathogen associated with tobacco target spot, but it was unexpected that it was also dominant on healthy leaves as well. By comparison, other studies of the tobacco leaf microbiome showed that the most abundant fungi were *Alternaria* and *Rhodotorula* for both healthy and brown spot infected tobacco leaves at the start of curing (Xiang et al., 2020a), and *Symmetrospora* and *Alternaria* for both healthy and brown spot infected tobacco leaves prior to application of Bordeaux mixture (Liu et al., 2022). Thus, the pathogen causing brown spot of tobacco, *Alternaria alternata* (Shew and Lucas, 1991), may also have been dominant in both diseased and healthy leaves, like *Thanatephorous* in this study. Only *Symmetrospora* was common as a dominant fungus between this study and previous ones. The diversity of fungi for healthy leaves was significantly higher than that of diseased leaves prior to azoxystrobin treatment indicating that infection by *R. solani* reduced leaf fungal diversity. Another study showed that infection of tobacco leaves by *A. alternata* reduced diversity of the fungal leaf microbiome (Liu et al., 2019). However, pumpkin leaves infected with *Podosphaera xanthii* showed a greater diversity in their fungal microbiomes than healthy leaves (Zhang et al., 2018).

### Changes in bacterial communities after azoxystrobin application

The diversity of the tobacco bacterial community significantly increased after azoxystrobin application for both healthy and diseased leaves. Similarly, application of another fungicide dimethachlon to healthy tobacco leaves increased the diversity of the bacterial community (Chen et al., 2021). This was proposed to be due to different species of bacteria being able to recolonize niches faster than others as the bacteria recovered from the effects of the fungicide. Also, application of the fungicide enostroburin to wheat leaves increased bacterial diversity believed to be due to a combination of the fungicide directly providing nutrients from emulsifiers and solvents in their formulation, releasing of nutrients from the killed fungi, and reducing microbial predation (Gu et al., 2010). In addition to direct antimicrobial effects of azoxystrobin, another effect on the microbiome could be surfactants included as spreaders in the fungicide formulation that could reduce adhesion of some of the bacterial genera onto the leaf allowing more types of bacteria to grow. There is strong evidence that association between microbial communities is related to their adhesion to the leaf surface (Monier and Lindow, 2004). Also azoxystrobin is a type of QoI fungicide, which is a class of fungicide able to prime plant defenses thus possibly indirectly affecting some endophyte populations (Skandalis et al., 2016).

By 18 dpt, the dominant bacterial genera of healthy leaves were *Pseudomonas* and *Methylobacterium*, whereas in diseased leaves, it was *Pseudomonas* and *Sphingomonas*. Similarly, after spraying dimethachlon on healthy tobacco leaves, the dominant bacterial genera were *Pseudomonas* and *Sphingomonas* (Chen et al., 2021). Among the ten most abundant bacteria, more changes in abundance over time were observed with diseased compared to healthy leaves. It could be that the fungicide has more of an effect on non-target organisms on diseased leaves as there is a considerable amount of the pathogen, which could be killed by the fungicide thus increasing niches for bacterial growth (Gu et al., 2010).

In this study, *Pseudomonas* showed a positive co-occurrence with *Pantoea* and a negative co-occurrence with *Lactobacillus*, whereas *Methylobacterium* showed a positive co-occurrence with *Aureimonas* but no negative co-occurrence with other bacterial genera. Thus, there is indication of positive and negative interactions between less dominant bacterial genera and the most dominant bacterial genera following azoxystrobin application. Positive co-occurrences could result from bacterial co-aggregation, co-colonization, co-survival and cross-feeding, whereas negative co-occurrences might result from bacterial toxins, selective detrimental changes to the microbial environment, or high niche overpopulation (Faust et al., 2012).

There were no significant differences between healthy and diseased leaves at any time point for any category of bacterial functions. There was also no significant differences for bacterial functions within healthy or diseased leaves over time. Thus, azoxystrobin appears to not to have shifted the overall functions of phyllosphere bacteria. In contrast, application of mancozeb to soil of potato significantly reduced functions for cellular processes and signalling, and significantly increased functions for amino acid metabolism of rhizosphere soil bacterial communities (Tang, 2020). However, rhizosphere soil is a very different environment than the leaf phylloplane or endophere, and thus the effect on the leaf may be quite different.

### Changes in fungal communities after azoxystrobin application

The diversity of the tobacco leaf fungal community significantly increased after azoxystrobin application to diseased leaves. Application of Bordeaux mixture to healthy tobacco leaves also increased the diversity of the fungal community, which was proposed to be due to the fungicide inhibiting the metabolism of only some of the fungal genera (Liu et al., 2022). Another factor could be in the interaction of bacteria and fungi after azoxystrobin treatment. Azoxystrobin can increase prokaryotic polysaccharide consumption and decrease prokaryotic vitamin biosynthesis and exchange, which can negatively affect the associated eukaryotic community (Lu et al., 2019). Thus, some fungi may have been more impacted by changes in bacterial metabolism. In addition, fungi differ in their sensitivity to azoxystrobin. For example, application of azoxystrobin inhibited mycelial growth of *Alternaria alternata*, *Alternaria* sp., *Bipolaris sorokiniana*, *Fusarium culmorum*, *Phoma glomerata* and *S. nodorum* ranging from 14.8% to 48.8% (Dzhavakhiya et al., 2012). Therefore, a possibility is that some of the rarer fungi may have been able to grow if they had greater resistance to azoxystrobin than some of the more sensitive dominant fungi, such as *Thanatephorous*. The decline in the population of the target spot fungus *Thanatephorous* in diseased leaves measured by DNA content was consistent with results of the culturable fungi isolated from treated diseased leaves and the reduced levels of target spot observed. The decline of *Thanatephorous* in healthy leaves indicates that azoxystrobin may be able to reduce the chances of future or latent target spot infections.

By 18 dpt, the dominant fungal genera of healthy leaves were *Thanatephorus*, whereas in diseased leaves, it was *Phoma*. However, after spraying Bordeaux mixture on healthy tobacco leaves, the dominant fungal genera were *Fusarium* and *Cercospora* (Liu et al., 2022). Among the ten most abundant fungal genera, four showed increased abundance over time in diseased leaves, and only the target spot pathogen *Thanatephorus* showed a progressive decline. Although a decline in *Thanatephorus* was also observed for healthy leaves, there were no progressive increases in any of the ten most abundant fungal genera. It could be that azoxystrobin had a progressive stimulatory effect on non-target fungi on diseased leaves as there is a considerable amount of the pathogen, which could be killed by the fungicide thus releasing nutrients from it hyphae and increasing the niches for use by other fungi (Perazzolli et al., 2014).

In this study, *Thanatephorus* showed no positive co-occurrence with any fungal genera but did have a negative co-occurrence with *Fusarium* and *Plectosphaerella*. *Phoma* showed a positive co-occurrence with *Symmetrospora* and *Aspergillus* but no negative co-occurrence. *Thanatephorous* was only fungal species not to show a positive co-occurrence with other fungal genera, which may because the damage it caused the leaves greatly perturbed the resident fungal microbiota (Hassani et al., 2018). Positive and negative co-occurrences between fungal genera in an environment could result from decreased or increased competition for nutrients or niches (Harcombe, 2010; Wintermute and Silver, 2010).

All the trophic modes of the fungi were observed on healthy and diseased leaves prior and post azoxystrobin treatment: pathotroph, pathotroph-saprotroph, pathotroph-saprotroph-symbiotroph, pathotroph-symbiotroph and saprotroph. On both healthy and diseased leaves, the relative abundance of pathotroph mode declined greatly between 0 and 3 dpt. This is mostly related to the decline in the abundance of the target spot pathogen *Thanatephorus* at that time, which comprised a large proportion of the fungal microbiome at 0 dpt. Other genera of possible tobacco pathogens that showed declines were *Cercospora* which could include *C. nicotianae* that causes frogeye spot of tobacco (Zhao et al., 2020), *Phoma* which could include *P. sorghina* that causes leaf spot of tobacco (Yuan et al., 2016), *Cladosporium* that could include *C. cladosporioides* that causes seed infections of tobacco (Wang et al., 2014) and *Fusarium* that could include *F. oxysporum* that causes fusarium wilt of tobacco (LaMondia, 2015). For the other three trophic modes, it was notable that there were large increases in abundance of all of them only on diseased leaves at 9 or 18 dpt, whereas they remained low on healthy leaves. This could be due to the decline in the relatively high abundance of *Thanatephorus* belonging to the pathotroph mode allowing later for more growth of fungi in the pathotroph-saprotroph, pathotroph-saprotroph-symbiotroph and pathotroph-symbiotroph modes as previously described. In no samples was there a significant difference in the abundance of a trophic mode between healthy and diseased leaves at a particular time point.

## Conclusions

Both healthy and target spot diseased leaves of *N. tabacum* had highly complex microbial communities prior to application of azoxystrobin with a higher diversity of fungi, but not bacteria, for healthy leaves compared to diseased leaves. Application of azoxystrobin to leaves had both a strong effect on the tobacco target spot pathogen and a semi-selective effect on the structure and diversity of tobacco leaf microbiome. Diversity of both bacteria and fungi increased with azoxystrobin application. Changes in bacterial abundance were greater with diseased than healthy leaves, whereas increased fungal abundance occurred only in diseased leaves. There were no changes in bacterial functions with azoxystrobin application in healthy and diseased leaves; however, fungal trophic modes were shifted with azoxystrobin application, but only in diseased leaves. Thus, both target and non-target effects of azoxystrobin were greater in diseased than healthy leaves, likely due to the prior perturbation of the micobiome produced by target spot infections as indicated by a higher fungal diversity for healthy leaves compared to diseased leaves before azoxystrobin application. Further study is needed to examine the length of time that the effects of azoxystrobin persist or if any of these changes have positive or detrimental effects on plant metabolism.

## Data availability statement

The datasets presented in this study can be found in online repositories. The names of the repository/repositories and accession number(s) can be found below: https://www.ncbi.nlm.nih.gov/, PRJNA779564 https://www.ncbi.nlm.nih.gov/, PRJNA779579.

## Author contributions

MS, HW, and CS contributed to conception and design of the study. MS, LX, TL, LC, XC conducted the experiment and collected the samples. MS, JL, TL and LX performed the analysis of samples. MS, JL and LX analyzed the data. MS wrote the first draft of the manuscript. HW and CS wrote sections of the manuscript. PG, HW, CS, JL, PG and LW revised the manuscript. All authors contributed to the article and approved the submitted version.
