# Supplementary material for: Effect of azoxystrobin on tobacco leaf microbial composition and diversity
Source: Front Plant Sci. 2023 Feb 1;13:1101039. doi: 10.3389/fpls.2022.1101039 (PMC9930646; doi:10.3389/fpls.2022.1101039)
Supplement: Supplementary file 1 [file DataSheet_1.docx]

**TABLE S1** Samples information.

| **Sampling group** | |  | **0 day before application** | **1 day after application** | **3 days after application** | **9 days after application** | **18 days after application** |
| --- | --- | --- | --- | --- | --- | --- | --- |
| **Healthy tissue** | **Sample No.** | | QJAJ11 | QJAJ21 | QJQJ31 | QJAJ41 | QJAJ51 |
|  |  |  | QJAJ12 | QJAJ22 | QJAJ32 | QJAJ42 | QJAJ52 |
|  |  |  | QJAJ13 | QJAJ23 | QJQJ33 | QJAJ43 | QJAJ53 |
|  | **Group No.** | | QJAJ1 | QJAJ2 | QJAJ3 | QJAJ4 | QJAJ5 |
| **Diseased tissue** | **Sample No.** | | QBAJ11 | QBAJ21 | QBAJ31 | QBAJ41 | QBAJ51 |
|  |  |  | QBAJ12 | QBAJ22 | QBAJ32 | —— | —— |
|  |  |  | QBAJ13 | QBAJ23 | QBAJ33 | —— | —— |
|  | **Group No.** | | QBAJ1 | QBAJ2 | QBAJ3 | QBAJ4 | QBAJ5 |

Note: Due to the interference of external factors, the diseased parts were not sampled repeat after 9 d and 18 d of post treatment.

**TABLE S2** Distribution of the top 10 dominant taxa of leaf microorganisms.

|  | **Endophytes** | | **references** | | **epiphytes** | | **references** | |  |
| --- | --- | --- | --- | --- | --- | --- | --- | --- | --- |
| **Bacteria** | | *Pseudomonas* | | Magnani et al. 2013 | | *Methylobacterium* | | Yang et al. 2021 | |
|  |  | *unidentified_Rhizobiaceae* | | Magnani et al. 2013 | | *Sphingomonas* | | Yang et al. 2021 | |
|  |  | *unidentified_Christensenellaceae* | | Magnani et al. 2013 | | *Pantoea* | | Yang et al. 2021 | |
|  |  | *Microbacterium* | | Paola et al. 2014 | | *Massilia* | | Yang et al. 2021 | |
|  |  | *Paenibacillus* | | Paola et al. 2014 | | *Aureimonas* | | Xiang et al. 2022 | |
| **Fungi** | *Pantospora* | | Feng et al. 2019 | | *Thanatephorus* | | Elliott et al. 2008 | |  |
|  | *Gomphidius* | | Feng et al. 2019 | | *Symmetrospora* | | Yang et al. 2021 | |  |
|  | *Phoma* | | Han et al. 2004 | | *Cercospora* | | Zhao et al. 2020 | |  |
|  |  | |  | | *Plectosphaerella* | | Xu et al. 2014 | |  |
|  |  | |  | | *Cladosporium* | | Yan et al. 2007 | |  |
|  |  | |  | | *Fusarium* | | Yang et al. 2021 | |  |
|  |  | |  | | *Sampaiozyma* | | Haelewaters et al. 2021 | |  |
